# Supplementary figures and images for: The extended day length promotes earlier flowering of bermudagrass
Source: PeerJ. 2022 Nov 16;10:e14326. doi: 10.7717/peerj.14326 (PMC9675341; doi:10.7717/peerj.14326)

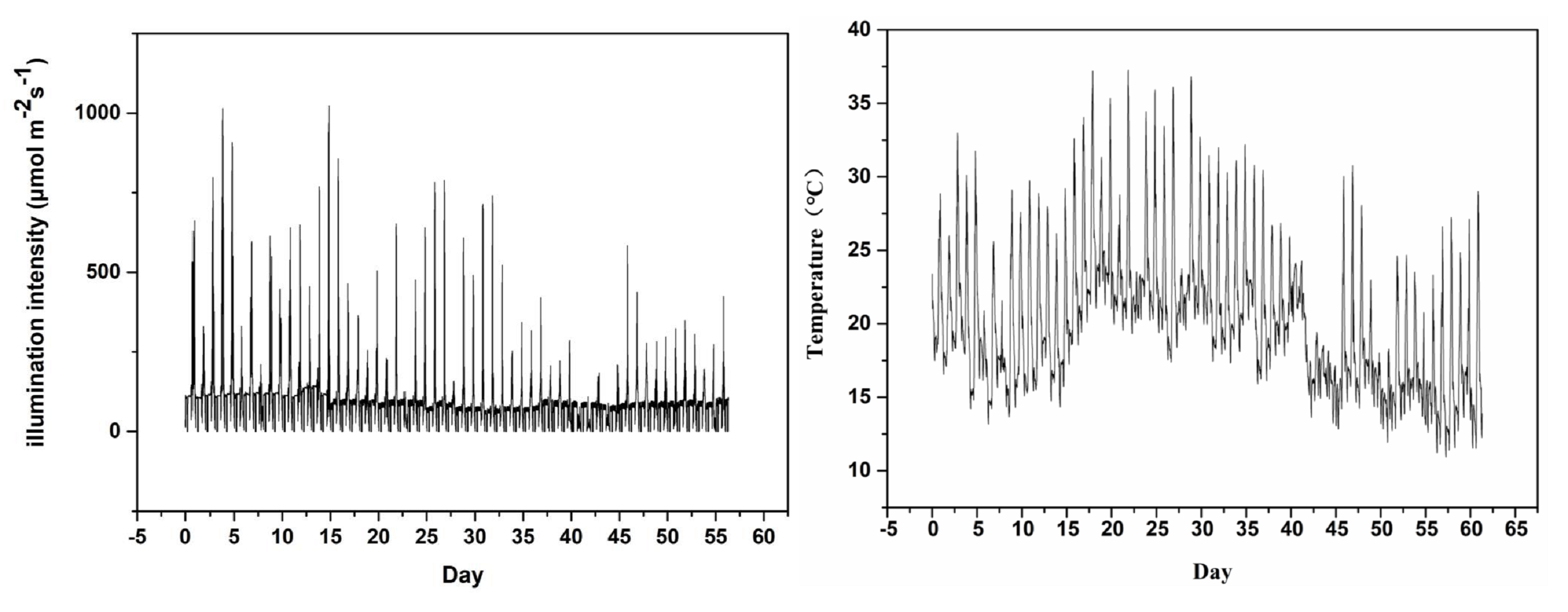

Supplement: Figure S1 — The temperature and light intensity of the greenhouse were recorded during the experiment (a) illumination intensity (b) temperature [file peerj-10-14326-s001.png]

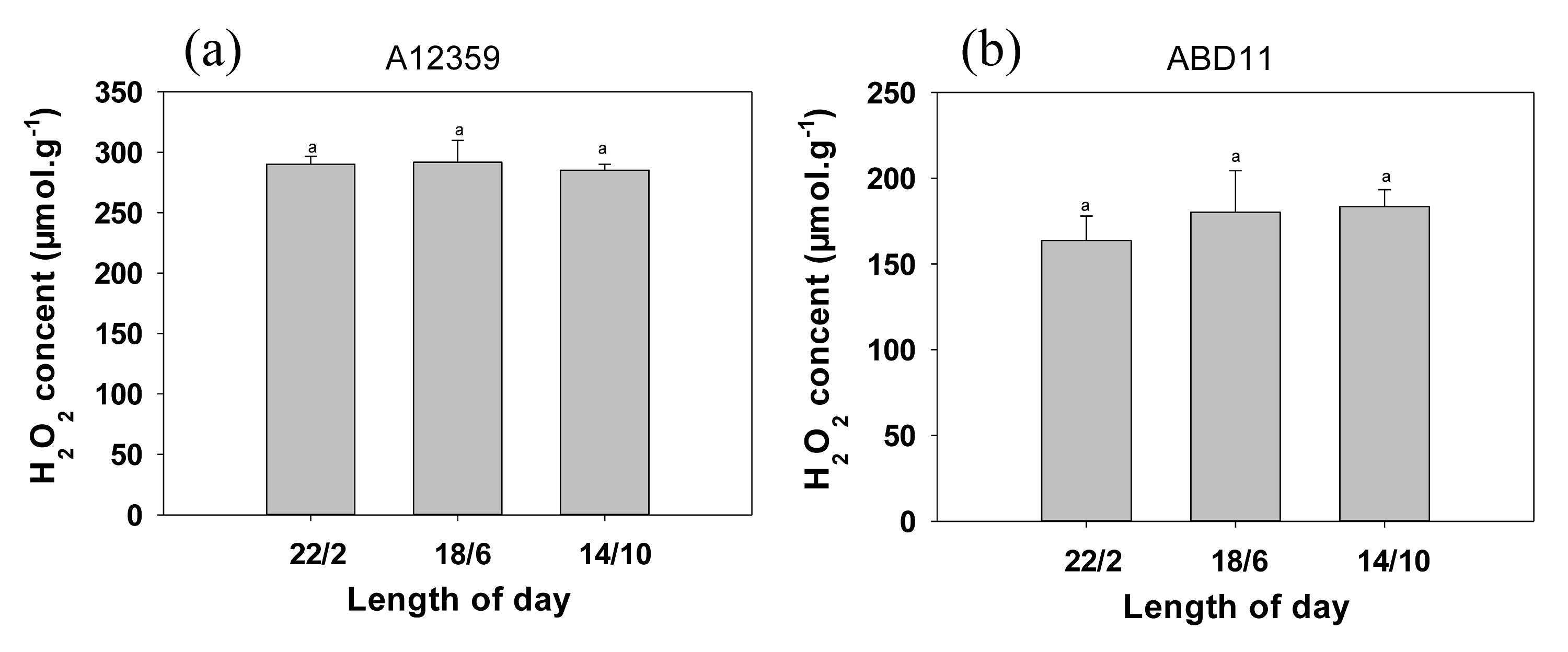

Supplement: Figure S2 — To verify the feasibility of the experimental design we measured the hydrogen peroxide content at the end of the experiment. The concent of H2O2 was measured using a commercially available kit (Nanjing Jiancheng Bioengineering Institute). For the determination of H2O2, the 0.1g of leaves of bermudagrass were ground in liquid nitrogen, dissolved in phosphate buffered solution (pH: 7.4, 0.1 mol/L), and then centrifuged for 10 min at 10000 g. The A405 was determined and the content of H2O2 was then calculated. Bar plots depicts the hydrogen peroxide content of the leaves at the end of the experiment. (a ) (b)Hydrogen peroxide content of leaves of A12359 and ABD11 under different application times (22/2, 18/6, 14/10). Each material is designed to be replicated three times. According to the SNK test at P < 0.05, different lowercase letters indicate significant differences between the three light application times at the same time. Whether it was A12359 or ABD11, there was no significant difference in hydrogen peroxide content between the longer and shorter light application times. These results suggested that prolonged light application time had no effect on ROS levels in bermudagrass. [file peerj-10-14326-s002.png]
