# Supplementary material for: The extended day length promotes earlier flowering of bermudagrass
Source: PeerJ. 2022 Nov 16;10:e14326. doi: 10.7717/peerj.14326 (PMC9675341; doi:10.7717/peerj.14326)
Supplement: Table S1 [file peerj-10-14326-s003.docx]

| **Supplementary Table 1**  **Two-way** **ANOVA to examine the effects of ‘Light application time’, ‘Week’ and**  **‘Light application time * Week’.** | | | | | |
| --- | --- | --- | --- | --- | --- |
|  | Variable | Factor | df | F | Sig |
| Fig2(A12359) | Plant hight | Week  Light application time  Week * Light application time | 6  2  12 | 42.114  256.353  12.765 | .000  .000  .000 |
| Fig2(A12359) | Fresh weight | Week  Light application time  Week * Light application time | 5  2  10 | 31.513  84.065  5.800 | .000  .000  .000 |
| Fig2(A12359) | Dry weight | Week  Light application time  Week * Light application time | 5  2  10 | 26.764  86.586  5.670 | .000  .000  .000 |
| Fig2(ABD11) | Plant hight | Week  Light application time  Week * Light application time | 6  2  12 | 67.217  91.512  4.014 | .000  .000  .000 |
| Fig2(ABD11) | Fresh weight | Week  Light application time  Week * Light application time | 5  2  10 | 43.604  108.444  6.363 | .000  .000  .000 |
| Fig2(ABD11) | Dry weight | Week  Light application time  Week * Light application time | 5  2  10 | 1.438  497.813  .818 | .000  .000  .000 |
| Fig3(A12359) | N | Week  Light application time  Week * Light application time | 5  2  10 | 5182.724  14501.002  1042.127 | .000  .000  .000 |
| Fig3(A12359) | P | Week  Light application time  Week * Light application time | 5  2  10 | 4822.937  14713.424  769.967 | .000  .000  .000 |
| Fig3(ABD11) | N | Week  Light application time  Week * Light application time | 5  2  10 | 3582.422  7707.885  978.506 | .000  .000  .000 |
| Fig3(ABD11) | P | Week  Light application time  Week * Light application time | 5  2  10 | 2271.239  3869.089  279.112 | .000  .000  .000 |
| Fig4(A12359) | Branch | Week  Light application time  Week * Light application time | 6  2  12 | 161.004  345.398  33.251 | .000  .000  .000 |
| Fig4(ABD11) | Branch | Week  Light application time  Week * Light application time | 6  2  12 | 104.876  103.987  6.925 | .000  .000  .000 |
